# The complete mitochondrial genome of *Neoris haraldi* Schawerda (Lepidoptera: Saturniidae)

**DOI:** 10.1080/23802359.2017.1372721

**Published:** 2017-09-08

**Authors:** Yu-Ying He, Xing Wang, Liu-Sheng Chen

**Affiliations:** a College of Agriculture, Shihezi University, Shihezi, Xinjiang, China;; b College of Plant Protection, Hunan Agricultural University, Changsha, Hunan, China

**Keywords:** *Neoris haraldi* Schawerda, mitochondrial genome, Saturniidae, evolutionary relationships

## Abstract

As an important insect pest on desert vegetation, *Neoris haraldi* has already brought great damage on *Populus euphratica* that was a key plant in the desert areas. Here, the complete mitochondrial genome (mitogenome) of *N. haraldi* has been sequenced with 15,383 bp in length. The mitogenome has a base composition of A (39.4%), T (40.3%), C (12.4%), and G (7.9%), and consists of 13 protein-coding genes (PCGs), 22 transfer RNA (tRNA) genes, 2 ribosomal RNA (rRNA) genes, and an A + T-rich region. The phylogenetic relationships among the saturniid species were (*Neoris haraldi*+ ((*Attacus atlas*+ (*Samia cynthia* + (*Samia canningi* +* Samia ricini*))) + ((*Eriogyna pyretorum* + *Saturnia boisduvalii*) + ((*Actias artemis* +* Actias selene*) + (*Antheraea assama*+ (*Antheraea frithi* + (*Antheraea pernyi* * Antheraea yamamai*))))))), which was supported by a high bootstrap value of 100% and a posterior probability of 1.00.

As an important insect pest on desert vegetation, *Neoris haraldi* Schawerda is widely distributed in Xinjiang, Shannxi, Gansu Provinces in China (Kereman et al. 2009). It has already brought great damage on *Populus euphratica* that is a key plant in the desert areas. Here, the mitochondrial genome of *N. haraldi* with its evolutionary position was sequenced and analysed. In September 2015, the female adult of *N. haraldi* was collected by Liu-Sheng Chen from Ku’erle city, Xinjiang Uygur Autonomous Region, and preserved in Shihezi University, Shihezi city, Xinjiang Uygur Autonomous Region, China.

The genomic DNA of *N. haraldi* was extracted and stored in Shihezi University for sequencing. The primers reported by Gu et al. (2016) were used for amplifying the complete mitogenome. The fragments were proof-read and assembled by the program Geneious 8.12 (Kearse et al. 2012), and the automatic annotation was done using by the online-program MITOS (Bernt et al. 2013). The complete mitogenomes of 12 statruniid species as ingroups, and one bombycid species and one sphingid species as outgroups were obtained from NCBI. The conserved regions of the putative amino acids from all 13 PCGs excluded the stop codons were filtrated by the software Gblock 0.91b with default settings. The phylogenetic tree was reconstructed by maximum likelihood (ML) with 1000 replications and Bayesian inference (BI) with running for 10,000,000 generations.

The entire mitogenome of *N. haraldi* has closed circular molecule with 15,383 bp in length (GenBank accession number MF664471), and a base composition of A (39.4%), T (40.3%), C (12.4%), and G (7.9%). It encoded 37 genes consisting of 13 PCGs, 22 tRNA genes, and two rRNA genes, as well as containing a putative A + T-rich region. Almost all of the PCGs started with ATN except *cox1* with CGA. Additionally, two PCGs (*cox1*, *cox2*) have a single stop codon T, and the other 11 PCGs have the complete stop codon TAA. A + T-rich region is located between *rrnS1* and *trnM* with 417 bp in length and has a high AT content of 90.9%. A conserved structure consisting of the motif ‘ATAGA’ was present in the downstream 19 bp of *rrnS1*, and three microsatellites ‘(AT)_5_’, ‘(AT)_8_’, and ‘(AT)_8_’ were located at the 163, 122,66 bp upstream of *trnM*, respectively.

The evolutionary relationships among the saturniid species were reconstructed, and the topological structures of the BI and ML trees were identical (Figure 1). The Saturniidae species were strongly supported as a monophyletic clade by the bootstrap value of 100% and the posterior probability of 1.00, and the phylogenetic position of *N. haraldi* among the family Saturniidae was *Neoris haraldi*+ ((*Attacus atlas*+ (*Samia cynthia*+ (*Samia canningi*+* Samia ricini*))) + ((*Eriogyna pyretorum*+* Saturnia boisduvalii*) + ((*Actias artemis*+* Actias selene*) + (*Antheraea assama*+ (*Antheraea frithi*+ (*Antheraea pernyi*+* Antheraea yamamai*)))))), which was supported as a monophyletic clade by a bootstrap value of 100% and a posterior probability of 1.00. The newly determined mitogenome is useful for understanding the evolution of the desert saturniid pest.

**Figure 1.**
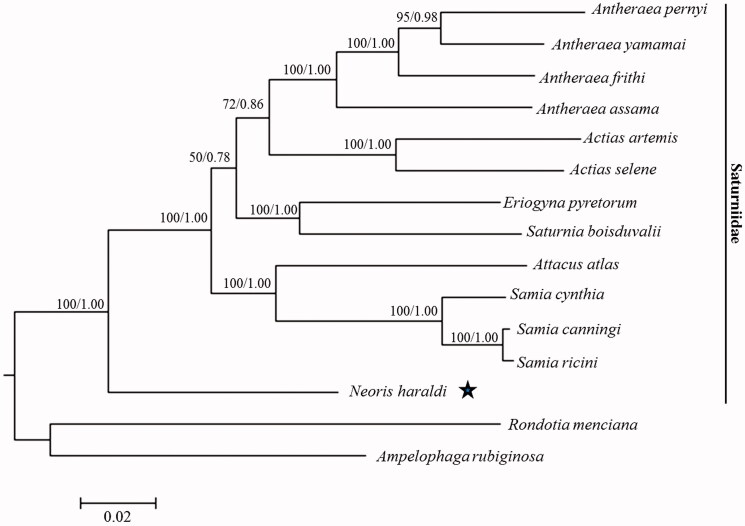
Maximum likelihood and Bayesian inference phylogram constructed using 13 PCGs of mitogenomes with partitioned models. Numbers above each node indicate the ML bootstrap support values and the BI posterior probability. All the species’ accession numbers in this study are listed as below: *Actias artemis* KF_927042, *Actias selene* NC_018133, *Ampelophaga rubiginosa* NC_035431, *Antheraea assama* NC_030270, *Antheraea frithi* NC_027071, *Antheraea pernyi* NC_004622, *Antheraea yamamai* NC_012739, *Attacus atlas* NC_021770, *Eriogyna pyretorum* NC_012727, *Neoris haraldi* MF664471, *Rondotia menciana* NC_021962, *Samia canningi* NC_024270, *Samia cynthia* KC812618, *Samia ricini* NC_017869, *Saturnia boisduvalii* NC_010613.
